# Effect of Fertilization and Row Spacing on the Performance of Nettle (*Urtica dioica* L.) Under Mediterranean Conditions

**DOI:** 10.3390/plants15101561

**Published:** 2026-05-20

**Authors:** Antonios Mavroeidis, Panteleimon Stavropoulos, Ioannis Roussis, Stella Karydogianni, George Papadopoulos, Stavroula Kallergi, Myrto Chatzitriantafyllou, Vasiliki Pachi, Dimitrios Beslemes, Evangelia Tigka, Ioanna Kakabouki, Dimitrios Bilalis

**Affiliations:** 1 Laboratory of Agronomy, Department of Crop Science, Agricultural University of Athens, Iera Odos 75, 118 55 Athens, Greece; stavropoulosp@aua.gr (P.S.); roussis@aua.gr (I.R.); karydogianni@aua.gr (S.K.); gpapadopoulos@aua.gr (G.P.); alinakallergi1@gmail.com (S.K.); myrtochatz16@gmail.com (M.C.); pahivasiliki@gmail.com (V.P.); i.kakabouki@aua.gr (I.K.); 2 Institute of Industrial and Forage Crops, Hellenic Agricultural Organization Dimitra, Theofrastou 1, 413 35 Larissa, Greece; dbeslemes@elgo.gr; 3 Department of Food Science and Nutrition, University of Thessaly, 43100 Karditsa, Greece; etig@uth.gr

**Keywords:** fertilization, Mediterranean, row spacing, *Urtica dioica*

## Abstract

The increasing demand for resilient and multifunctional crops in the Mediterranean region has renewed interest in *Urtica dioica* L. as a potential alternative crop. This study evaluated the combined effects of fertilization and row spacing on the growth, yield, and nitrogen use efficiency of nettle in Athens, Greece. A split-plot experimental design was employed in a three-year experiment, with three fertilization treatments (C = control, U = urea, and I = urea with urease inhibitor) and two different row spacings (D1 = 30 cm × 20 cm, and D2 = 50 cm × 20 cm). Agronomic traits, seed yield, nitrogen content, vegetation indices (NDVI), chlorophyll content (SPAD), and nitrogen efficiency indices were assessed. Fertilization significantly enhanced plant performance, with the application of I consistently producing the highest values for plant height (increased by 10–30%), biomass (increased by 10–20%), and seed yield (increased up to 30%) compared to C. Row spacing influenced crop performance, with D2 favoring plant height (up to 9% compared to D1), while D1 generally increased biomass production per unit area (up to 20% compared to D2). Nitrogen-related indices (NUE, NAE, and NUtE) were markedly improved under fertilized treatments, particularly when I was applied (up to 20%, 100%, and 19% compared to U). NDVI and SPAD values were also influenced by fertilization and row spacing at early growth stages. The findings demonstrate that both factors play critical roles in optimizing nettle cultivation under Mediterranean conditions, highlighting the importance of integrated agronomic management practices.

## 1. Introduction

The interest regarding the cultivation of novel crops in Mediterranean regions has been recently increasing [1]. The Mediterranean Basin is regarded as one of the most vulnerable regions to climate change [2]. The rising temperatures, intensive droughts, and frequent extreme weather phenomena have a significant negative impact in agriculture, affecting crop performance and yields [3]. The incorporation of resilient crops with high acclimatization potential to the region’s agrifood systems has been proposed as a means to adapt to climate change’s adverse effects on agriculture [1]. A promising crop that fits this narrative is *Urtica dioica* L. [4] (commonly known as stinging nettle), a perennial herbaceous plant belonging to the Urticaceae family [5]. It is a cosmopolitan weed, widely distributed across temperate regions of Europe, Asia, North Africa, and North America [6]. The species is characterized by vigorous vegetative growth, high ecological plasticity, and the presence of stinging trichomes that deter herbivory [7,8,9].

Even though nettle is regarded as a weed in conventional agricultural systems [10], it has been recognized for its significant nutritional, medicinal, and industrial value [4]. Traditionally, various parts of the plant have been used in human and animal nutrition due to their high protein content, essential amino acids, vitamins, and minerals [11,12,13]. In addition, it contains bioactive compounds that are widely employed in herbal medicine for their anti-inflammatory, antioxidant, and diuretic properties [14], while fibers derived from its stems have potential applications in the textile industry [15]. Due to its multiple applications, *U. dioica* is simultaneously considered both a weed and a minor/alternative crop [16]. This dual status limits the development of standardized cultivation practices, as nettle production has historically relied on wild harvesting rather than managed agricultural systems [17]. However, increasing demand for natural products, functional foods, and sustainable raw materials has stimulated interest in the domestication and agronomic optimization of nettle [17,18]. Transitioning nettle from a wild or underutilized species to a major crop presupposes a thorough assessment of the factors influencing its growth, biomass production, and overall performance under different environmental conditions [19].

Among agronomic practices, fertilization and row spacing are key management factors that strongly affect crop performance in nettle [4]. Fertilization influences nutrient availability and uptake, which in turn affects vegetative growth, biomass accumulation, and biochemical composition [20]. *U. dioica* is regarded as a nitrophilic species and it is known to respond positively to nitrogen (N) [21]; however, a wide range of fertilization regimes have been proposed as optimal for its cultivation [4]. Row spacing also affects nutrient uptake and plays a critical role in determining light interception, intra-specific competition, and canopy structure [22]. Optimal spacing can maximize yield, while overly dense or sparse stands may reduce crop performance or total biomass production [23]. Despite the suggested importance of fertilization and spacing, information regarding their combined effects on nettle growth under Mediterranean conditions remains limited. The aim of the present study was to evaluate the effect of three different fertilization regimes (unfertilized control, application of urea, and application of urea with urease inhibitors) and two different row spacing arrangements (30 cm × 20 cm, and 50 cm × 20 cm) on *U. dioica* under Mediterranean conditions.

## 2. Results

### 2.1. Agronomic Traits and Seed Yield

According to the results of the present study, fertilization had a statistically significant effect on the agronomic traits and the seed yield of *U. dioica* (Table 1). In most cases, the best results were reported in I and the lowest values were observed in C. Plant height was notably affected by fertilization. Particularly, U and I increased average height by 4–18% and 10–30% respectively, when compared to C. Similarly, treatments U and I significantly increased fresh and dry weight, and the seed yield. In both D1 and D2, U increased plant fresh weight ha^−1^ by 8–11% and I increased it by 17–21% (compared to C). Regarding the plants’ dry weight, the application of U and I resulted in an increase of 6–20% and ≈10–30% respectively compared to C (Table 1). Seed yield per ha was statistically significantly increased by the application of fertilization, with the best results reported in I. In this treatment, fertilization increased average seed yield by even as much as three-fold. However, in D2 of the second experimental year I reported the lower yields, by 30% and 47% compared to the respective ones of C and U.

Row spacing had also a significant impact on the results. In most measurements, the results regarding plant height reported from D2 were significantly higher than the ones in D1, as plant height was increased by 4–8%, 2–6%, and 2–4% in C, U, and I respectively (Table 1). On the contrary, fresh and dry weight were notably higher in D2. During the first and the second experimental years, fresh weight per ha was higher in D1 by 16–23% (compared to D2), regardless of fertilization treatments. However, in the third year D2 reported higher fresh weight values by 16–17%. The same pattern was observed also in dry weight. In the first and second experimental years, plants’ dry weight was significantly higher in D1 (approximately by 17–20% compared to D2), and in the third, D2 plants reported a significantly higher dry weight (approximately by 15% compared to D2), regardless of fertilization treatments.

Additionally, the factor “Year” significantly affected crop performance and yield (Table 1). Average plant height, fresh weight, and dry weight increased significantly over time, with the highest values for each trait recorded in the third experimental year. It should be noted that, in most cases, the rate of increase in plant height and weight was not maintained consistently across the three experimental years; rather, it declined with each successive year. A case in point is plant height in ID1. In the first experimental year, treatment I in D1 increased average plant height by 32% compared to C. However, in the second and third years, this increase was reduced to 16% and 10%, respectively. Average seed yield also exhibited an overall increasing trend; however, significant fluctuations were observed. For example, average yield in CD2 increased from 17 kg ha^−1^ to 48 kg ha^−1^ in the second year and then decreased to 24 kg ha^−1^ in the third year.

### 2.2. NDVI and SPAD

**Figure 1:**
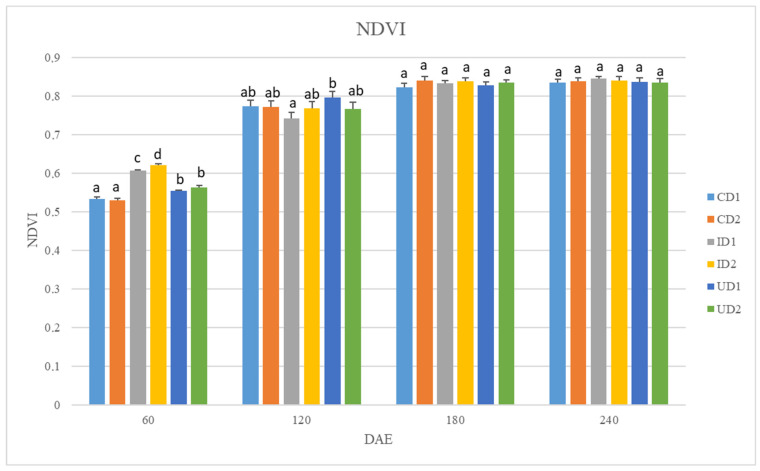
NDVI values 60, 120, 180, and 240 days after plant emergence (DAE). Lower case letters a, b, c, and d correspond to statistically significant differences among treatments. Treatments include: CD1, unfertilized control and row spacing 30 cm × 20 cm; CD2, unfertilized control and row spacing 50 cm × 20 cm; ID1, application of 200 kg N in the form of urea 46-0-0 with urease inhibitors and row spacing 30 cm × 20 cm; ID2, application of 200 kg N in the form of urea 46-0-0 with urease inhibitors and row spacing 50 cm × 20 cm; UD1, application of 200 kg N in the form of urea 46-0-0 and row spacing 30 cm × 20 cm; UD2, application of 200 kg N in the form of urea 46-0-0 and row spacing 50 cm × 20 cm.

Average NDVI values reported significant differences only 60 and 120 DAE and were affected by both fertilization and row spacing, though they were unaffected by the factor “Year”. The highest NDVI values 60 DAE were observed in I and particularly in D2 (averaging at 0.62) and the lowest in C (0.53). The average NDVI values 120 DAE reported statistically significant differences only between I and U in D1, with the latter being increased by 7% (Figure 1).

**Figure 2:**
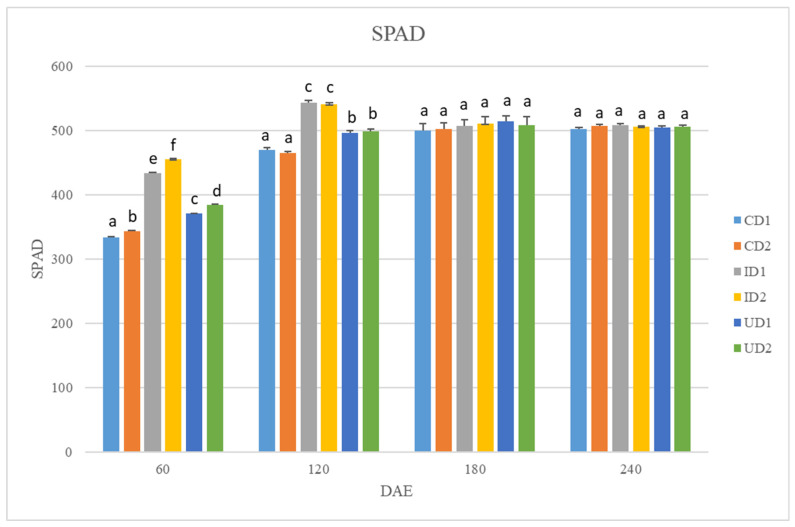
SPAD values 60, 120, 180, and 240 days after plant emergence (DAE). Lower case letters a, b, c, d, e, and f correspond to statistically significant differences among treatments. Treatments include: CD1, unfertilized control and row spacing 30 cm × 20 cm; CD2, unfertilized control and row spacing 50 cm × 20 cm; ID1, application of 200 kg N in the form of urea 46-0-0 with urease inhibitors and row spacing 30 cm × 20 cm; ID2, application of 200 kg N in the form of urea 46-0-0 with urease inhibitors and row spacing 50 cm × 20 cm; UD1, application of 200 kg N in the form of urea 46-0-0 and row spacing 30 cm × 20 cm; UD2, application of 200 kg N in the form of urea 46-0-0 and row spacing 50 cm × 20 cm.

Similarly to the NDVI values, SPAD values were affected by fertilization and row spacing only 60 and 120 DAE (Figure 2). Once again, I reported the highest values 60 DAE and C the lowest. In fact, in D1 60 DAE I and U increased SPAD values by 30% and 11% (compared to C) and in D2 by 32% and 12%, respectively. Moreover, D2 reported significantly higher values compared to D1. Row spacing did not have a significant impact on SPAD values 120 DAE. However, I and U increased them by approximately 15% and 6% respectively, compared to C.

### 2.3. N Yield and Crop Performance Indicators

**Figure 3:**
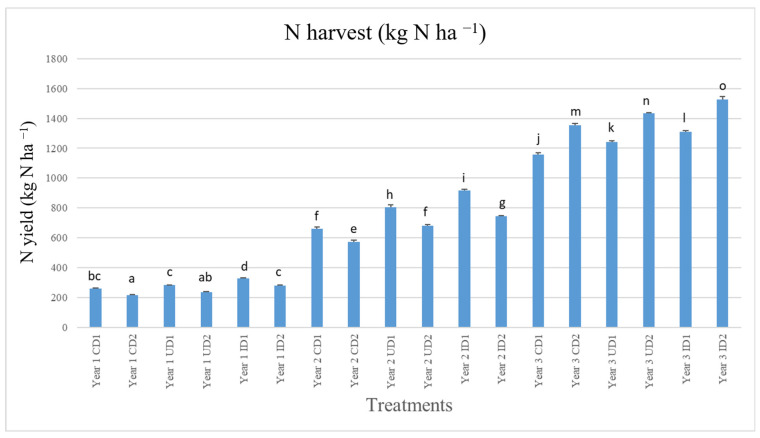
Annual N yield per year, fertilization treatment, and row spacing arrangement. Lower case letters a, b, c, d, e, f, g, h, i, j, k, l, m, n and o correspond to statistically significant differences among treatments. Treatments include: CD1, unfertilized control and row spacing 30 cm × 20 cm; CD2, unfertilized control and row spacing 50 cm × 20 cm; ID1, application of 200 kg N in the form of urea 46-0-0 with urease inhibitors and row spacing 30 cm × 20 cm; ID2, application of 200 kg N in the form of urea 46-0-0 with urease inhibitors and row spacing 50 cm × 20 cm; UD1, application of 200 kg N in the form of urea 46-0-0 and row spacing 30 cm × 20 cm; UD2, application of 200 kg N in the form of urea 46-0-0 and row spacing 50 cm × 20 cm. Year 1, 2, and 3 correspond to 2021, 2022, and 2023 respectively.

Total aboveground N, or N yield, was significantly affected by both row spacing and fertilization, as well as the experimental year. In fact, the interaction of these three factors was found statistically significant (*p*-Value < 0.05). N yields in the first experimental year ranged between 216 and 328 kg N ha^−1^, and between 573 and 916 and 1158 and 1526 kg N ha^−1^ in the second and third year, respectively (Figure 3). Overall, highest N yields were reported in I and D2 and lowest in C and D1.

All of the indicators assessed in the present study were affected by fertilization. NUE and NAE were significantly higher in I compared to U. NUE values in I were up to three-fold higher compared to the respective ones of U in both D1 and D2. Likewise, the application of I resulted in an increase of 65–150% in NAE values, compared to U (Table 2). The lowest NUtE values were noted in C, where they ranged between 1.2 and 2.2. Treatments U and I increased the respective NUtE values by approximately 15–30% and 25–50%, though in the second experimental year, the highest NUtE values were noted in C. This was also the case with HI as, throughout the experiment, the highest values were reported in C, whereas I reduced them by up to 0.1%.

Lastly, row spacing affected the values of all indicators (NUE, NUtE, NAE, and HI) (Table 2). Notably though, there was no clear positive or negative correlation between row spacing and the indicators. For instance, in U, NUE values were significantly higher in D2 in the first experimental year and in D1 in the third experimental year, while no significant differences were observed in the second year (Table 2). Similar patterns were noted also in NUtE, NAE, and HI values.

## 3. Discussion

Based on the findings of the present study, both fertilization and row spacing have a significant impact on the performance of *U. dioica*. Fertilization in particular was found positively correlated with all agronomic traits (plant height and weight) and the seed yield (Table 3). This was partially anticipated as the literature suggests that the application of 200 kg N ha^−1^ improves the yield components of nettle [24]. In a study by Radman et al. [25], authors applied the same N fertilization rates (in the form of calcium ammonium nitrate) in nettle and reported a similar increase in fresh weight. Grevsen et al. [26] observed that the application of N in *U. dioica* increases fresh weight by up to five-fold, though in their study fertilization rates were significantly higher (300 kg N ha^−1^). Several additional works have reported coinciding results that validate the positive response of the crop to N fertilization [27,28]. Τhis response can be attributed to the N fertilization-induced improvement of the plants’ photosynthetic activity [29,30]. As a result, plants possess more vigor [31], establish a better canopy [32], and increase their biomass [33].

However, the application of N fertilizers with inhibitors in *U. dioica* has not been thoroughly studied. In the present study, both U and I significantly increased plant height, though in most cases the latter was superior (Table 1). Inhibited fertilizers reduce N losses and sustain its availability throughout the crop’s different growth stages [34]. The literature suggests that N is essential for chlorophyll formation, cell division and elongation, and protein synthesis [35,36]. Therefore, inhibited fertilizers that prolong N availability can improve photosynthetic capacity (due to higher chlorophyll content) and increase plant height [36,37]. Additionally, sustained ammonium/nitrate supply supports steady stem elongation, thus potentially improving plant height [38]. The beneficial effect of inhibited fertilizers on the photosynthetic capacity of nettle plants would also account for the increased fresh and dry weight that was observed in I. Improved photosynthetic rates and N availability can prolong the vegetative stage in a crop’s growth cycle [39]. This often translates to greater production of vegetative organs, including increased leaf area and shoot elongation, and therefore higher above-ground biomass [40].

The above-mentioned hypotheses are reinforced by the NDVI and SPAD values that were reported in the present study. The significantly higher NDVI and SPAD values recorded during the vegetative stage of the crop (60 DAS) in I indicate a greater chlorophyll content in the leaves and enhanced photosynthetic activity. High SPAD and NDVI values in the early growth stages have been correlated with improved performance in studies conducted on other crops. Studies regarding field crops such as wheat, rice, and maize have established a correlation between in-season NDVI values and yields, as early NDVI values could reflect canopy development and biomass accumulation [41,42,43]. Similarly, studies suggest that SPAD could function as an early-stage yield indicator [44,45].

Seed yield responded positively to fertilization, and in particular to treatment I. Research regarding seed production and optimization of seed yield in *U. dioica* is admittedly limited [46]. In the present study, seed yields reached an average of 50 kg ha^−1^, in contrast to other available studies. In their work, Kosolapov et al. [47] reported seed yields that could exceed 100 kg ha^−1^, despite the absence of fertilization. The significant difference in the yield between their work and the present study could probably be attributed to the different pedoclimatic conditions and/or the use of different cultivars. Regardless of the seed yield, the elevated NUE, NUtE, and NAE values in I indicate optimized N efficiency and utilization. Once again, this further validates the improved photosynthesis hypothesis as improved N efficiency and utilization has been correlated with enhanced photosynthetic activity [48,49]. Even though there are no available studies that have evaluated these indicators in nettle cultivation, according to the literature, inhibited fertilizers have reported improved values of these indicators in wheat, maze, rice, and more [50,51,52]. It should be noted that in our study, fertilization and HI values were negatively correlated. This was probably due to the disproportionate increase in plants’ biomass compared to the seed yield.

The N yield results conform to the positive impact of I on the performance of the crop. Our results dictate that I increased significantly N yield, in accordance with the findings of Mavroeidis et al. [4]. As mentioned above, inhibited fertilizers prolong N availability and improve N supply [34]. At the root level, this increased external N availability can induce morphological and physiological responses that favor greater N uptake [53]. In some cases, plants exposed to higher N supply often exhibit increased root surface area and altered root architecture, improving their capacity to explore the soil for nutrients [54,55,56]. More importantly, the activity and expression of membrane-bound N transport systems can be upregulated, enabling plants to absorb larger quantities of available N when supply is abundant [57]. This regulation ensures that fertilized plants can capitalize on increased soil N by elevating their uptake rates. Additionally, N fertilization has been proposed to increase the plant’s capacity to assimilate inorganic N into organic forms [58]. This leads to greater accumulation of N-containing compounds such as amino acids, proteins, and structural components in plant tissues, thus improving N yield. It should be noted that in the present study, N yield was not solely affected by fertilization, as on average it increased each crop season. According to Mavroeidis et al. [4], this could possibly be attributed to the better establishment of the crop that improved N uptake and assimilation. Lastly, row spacing had a significant yet unconclusive effect on N yield.

Even though row spacing was not found positively or negatively correlated with any agronomic trait (Table 3), it had a significant impact on both plant height and weight (Table 1). The height of plants in D2 was significantly increased, thus implying that the lower plant density favored stem elongation. This coincides with the published literature that proposes larger row spacing in nettle cultivated for fiber production [22]. Fiber production presupposes larger stems [59], and lower plant densities can achieve it via improved nutrient uptake and use efficiency [60]. NUtE values recorded in D2 support this hypothesis, though NUE and NAE values did not follow a similar pattern. Additionally, higher SPAD and NDVI values in D2 60 DAS suggest increased photosynthetic activity that could result in plant growth and enhanced height. Contrasting to height, fresh and dry weight per ha was notably greater in D1. This finding probably indicates that the higher number of nettle plants per ha compensates for the superior N use efficiency in D2; hence, the total biomass ha^−1^ in D1 surpassed the respective one of D2. Overall, proposed row spacing in *U. dioica* varies from 20 to 75 cm [17,61]. Based on our findings, D1 in combination with the application of 200 kg N ha^−1^ in the form of inhibited fertilizers yields sufficient above-ground biomass, within the range reported in the literature. However, the findings regarding the effect of row spacing on seed yield were inconclusive and did not favor either D1 or D2.

## 4. Materials and Methods

### 4.1. Study Site, Environmental Conditions, and Soil Characterization

**Figure 4:**
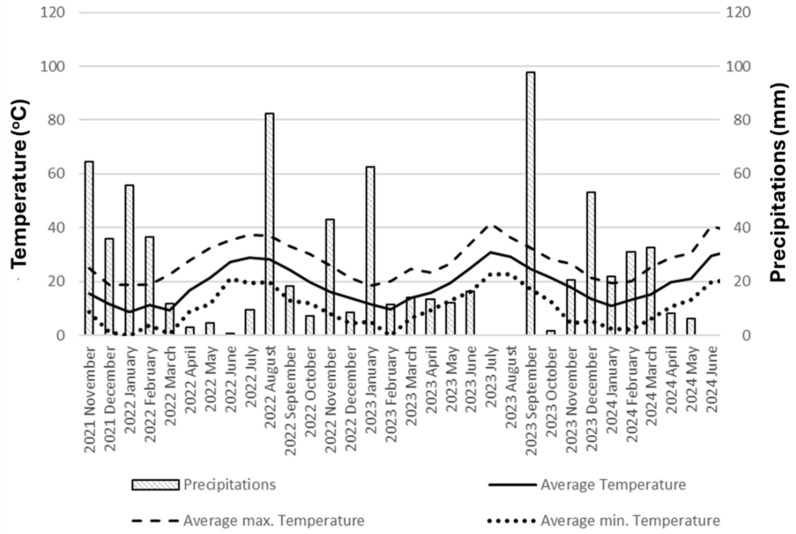
Weather data throughout the duration of the experiment.

A three-year field study (2021–2024) was carried out at the experimental field of the Laboratory of Agronomy, Agricultural University of Athens (37°59′ N, 23°42′ E). The soil at the experimental site was classified as clay loam, with a slightly alkaline pH and relatively low organic matter content. Climatic conditions were characteristic of a Mediterranean environment, with high summer temperatures reaching up to 40 °C and autumn precipitation exceeding 80 mm per month. The physicochemical characteristics of the soil are summarized in Table 4, while climatic conditions, including mean air temperature and mean precipitation, are illustrated in Figure 4. Soil sampling was conducted at a depth of 0–30 cm for analytical purposes. Meteorological observations were obtained from an on-site automated weather station (Davis VantagePro2, Davis Instruments Corporation, Hayward, CA, USA).

### 4.2. Crop Establishment, Experimental Design and Treatments

In November 2021, seeds of *Urtica dioica* (Agrogen S.A., Athens, Greece) were sown in 0.4 L plastic containers (10.5 × 8 cm) and maintained under greenhouse conditions at the Laboratory of Agronomy until emergence. The pots were filled with soil originating from the experimental field, and five seeds were manually sown per pot. Following emergence, seedlings were thinned to one per pot and subsequently transplanted to the field (20 DAS). The experiment was arranged in a split-plot design, with fertilization treatments assigned to main plots. These treatments included: (i) an unfertilized control (C), (ii) urea application (U), and (iii) urea supplemented with a urease inhibitor (I). Sub-plots corresponded to two different row spacing arrangements: 20 cm × 30 cm (D1) and 20 cm × 50 cm (D2). Each treatment combination was replicated four times. Main plots covered 50 m^2^, while sub-plots occupied 25 m^2^, resulting in a total experimental area of approximately 700 m^2^, including buffer zones between plots. Nitrogen fertilization in the U and I treatments was applied as a basal dose during the first growing season, whereas in subsequent years it was broadcast after seedling emergence. Nitrogen was applied at a rate of 200 kg N ha^−1^ using either urea (46-0-0; EUROCHEM HELLAS S.A., Athens, Greece) or urea amended with the urease inhibitor N-(2-nitrophenyl) phosphoric triamide (2-NPT; 0.035%). Weed control was performed manually as needed. The crop was grown under rainfed conditions without supplemental irrigation.

### 4.3. Growth and Yield Measurements

Crop performance was evaluated at harvest, which occurred 240 days after seedling emergence (240 DAS). Measured parameters included plant height, fresh biomass, and dry biomass. Seed harvesting and handling followed established propagation protocols for *U. dioica* [62], with seeds collected manually. For plant sampling, three quadrats (0.25 m^2^ each) were randomly placed within each sub-plot, and all plants within each quadrat were cut at ground level. Plant height was recorded immediately after sampling, and fresh weight was determined. For dry biomass estimation, samples were oven-dried at 80 °C for 72 h. The dried material was subsequently ground into a fine, homogeneous powder for nitrogen analysis.

### 4.4. Physiological Measurements

Total nitrogen content was determined using the Kjeldahl method [63] with a Kjeltec 2300/8400 Analyzer Unit (Foss Tecator AB, Höganäs, Sweden). Approximately 0.5 g of each sample was digested in concentrated H_2_SO_4_ with Se–CuSO_4_ catalysts at 420 °C, followed by distillation and titration with standardized 0.1 N HCl. The same analytical procedure was applied to seed samples collected annually. The seed and plant N content was then used for the estimation of the Nitrogen Use Efficiency (NUE) (1), the Nitrogen Utilization Efficiency (NUtE) (2), and the Nitrogen Harvest Index (NHI) (3) indices. The seed yield and the dry weight of the plants were used to estimate the Harvest Index (HΙ) (4). These indicators were estimated based on the following equations [34]:

NUE = [Aboveground N content in fertilized plants (kg ha^−1^) − Aboveground N content in control plants (kg ha^−1^)]/kg of applied N ha^−1^ (1)

NUtE = Seed yield (kg ha^−1^)/Aboveground N content (kg ha^−1^) (2)

NAE = Seed N content (kg ha^−1^)/Aboveground N content (kg ha^−1^) (3)

HI = Seed weight (kg ha^−1^)/Aboveground dry weight (kg ha^−1^) + seed weight (kg ha^−1^) (4)

N yield = Aboveground N content (kg ha^−1^) (5)

Normalized Difference Vegetation Index (NDVI) measurements were obtained using a GreenSeeker HCS-250 handheld active sensor (Trimble Agriculture, Sunnyvale, CA, USA). NDVI was calculated according to the following equation:

NDVI = (NIR − Red)/(NIR + Red)(6)

where NIR is the reflectance in the near-infrared band and Red is the reflectance in the red band. The sensor was positioned 60 cm above the plant canopy. Leaf chlorophyll content was assessed using a SPAD−502 m (Konica Minolta, Tokyo, Japan). Measurements were conducted on five randomly selected plants per sub-plot, with ten readings per plant, 60, 120, 180, and 240 days after plant emergence (DAE).

### 4.5. Statistical Analysis

Data were tested for normality and analyzed using analysis of variance (ANOVA) with SPSS software (version 22.0, IBM Corp., Armonk, NY, USA). Mean differences were compared using Tukey’s honestly significant difference (HSD) test at a significance level of *p* ≤ 0.05. Correlation analysis was also performed to evaluate relationships among the measured variables.

## 5. Conclusions

The results of the present study clearly demonstrate that *U. dioica* can perform adequately under Mediterranean conditions, with fertilization and row spacing emerging as key factors influencing crop performance, yield, and nitrogen use efficiency. Among the tested fertilization strategies, the application of urea in combination with a urease inhibitor proved to be the most effective, consistently enhancing plant height, biomass accumulation, and nitrogen-related efficiency indices. This treatment significantly outperformed both the unfertilized control and the sole urea application, indicating that the use of urease inhibitors can improve nitrogen availability and uptake, thereby increasing overall crop productivity. Row spacing also played an important, though more variable, role. Higher plant density generally promoted greater biomass production per unit area, particularly during the early years of the experiment, while lower density favored increased plant height. However, the effect of row spacing on yield and nitrogen efficiency indices was not always consistent. Overall, the combination of urea with a urease inhibitor and appropriate row spacing can substantially enhance nettle productivity and resource use efficiency. These findings contribute to the development of standardized agronomic practices for nettle cultivation and support its potential as a sustainable alternative crop in Mediterranean agroecosystems. Future research should focus on long-term performance, optimization of fertilization rates, and the interaction of agronomic practices with environmental stress factors.

## Figures and Tables

**Table 1:** Average plant height, fresh weight, dry weight, and seed yield ha^−1^.

Fertilization Treatments	Row Spacing	Height (cm)	Fresh Weight (tonnes ha^−1^)	Dry Weight (tonnes ha^−1^)	Seed Yield (kg ha^−1^)
2021					
C	D1	39.62 ± 0.39 a	3.62 ± 0.06 a	1.60 ± 0.01 a	26.44 ± 0.74 a
	D2	42.78 ± 0.47 A	3.06 ± 0.03 A	1.32 ± 0.01 A	17.79 ± 0.35 A
U	D1	46.68 ± 0.46 b	4.04 ± 0.04 b	1.71 ± 0.01 b	33.12 ± 0.74 b
	D2	49.54 ± 0.79 B	3.39 ± 0.03 B	1.42 ± 0.02 B	22.66 ± 0.81 B
I	D1	52.60 ± 0.69 c	4.38 ± 0.02 c	1.89 ± 0.02 c	43.42 ± 1.27 c
	D2	54.79 ± 0.42 C	3.62 ± 0.02 C	1.61 ± 0.03 C	47.88 ± 0.74 C
2022					
C	D1	57.43 ± 0.70 a	8.80 ± 0.05 a	3.95 ± 0.07 a	26.12 ± 0.38 a
	D2	59.56 ± 0.31 A	7.22 ± 0.04 A	3.40 ± 0.06 A	48.71 ± 1.55 B
U	D1	61.90 ± 0.35 b	9.58 ± 0.05 b	4.72 ± 0.08 b	31.02 ± 0.77 b
	D2	64.59 ± 0.37 B	7.87 ± 0.05 B	3.98 ± 0.02 B	54.82 ± 0.73 C
I	D1	66.62 ± 0.75 c	10.45 ± 0.05 c	5.27 ± 0.04 c	58.17 ± 0.74 c
	D2	68.79 ± 0.35 C	8.49 ± 0.05 C	4.25 ± 0.03 C	37.18 ± 0.89 A
2023					
C	D1	70.54 ± 0.35 a	11.11 ± 0.08 a	6.60 ± 0.06 a	33.86 ± 0.61 a
	D2	72.61 ± 0.35 A	13.03 ± 0.12 A	7.67 ± 0.05 A	24.27 ± 0.82 A
U	D1	73.70 ± 0.24 b	12.43 ± 0.11 b	7.02 ± 0.02 b	48.69 ± 0.55 b
	D2	75.54 ± 0.33 B	14.53 ± 0.13 B	8.04 ± 0.01 B	36.07 ± 2.42 B
I	D1	77.77 ± 0.35 c	13.49 ± 0.13 c	7.28 ± 0.02 c	44.60 ± 3.10 b
	D2	79.48 ± 0.45 C	15.81 ± 0.07 C	8.37 ± 0.07 C	48.50 ± 4.59 C
FYEAR (A)		4867.85 ***	26,610.22 ***	29,445.36 ***	70.04 ***
FFERTILIZATION (B)		597.86 ***	693.75 ***	402.25 ***	251.99 ***
FROW SPACING (C)		104.40 ***	8.02 **	0.01 ns	1.22 ns
FA × B		17.30 ***	88.19 ***	46.95 ***	9.08 ***
FB × C		0.41 ns	0.26 ns	4.96 **	1.09 ns
FA × C		1.21 ns	1112.91 ***	756.97 ***	37.55 ***
FA × B × C		0.20 ns	3.87 ns	5.42 *	40.19 ***

Means within a column followed by the different letters are significantly different at *p* = 0.05. Lower case letters a, b, and c correspond to statistically significant differences among fertilization treatments in D1, and uppercase letters A, B, and C correspond to statistically significant differences among fertilization treatments in D2. F-test ratios are from ANOVA, whereas ns, *, **, and *** correspond to no statistical significance, statistically significant at a level of *p* < 0.05, statistically significant at a level of *p* < 0.01, and statistically significant at a level of *p* < 0.001 respectively.

**Table 2:** Average NUE, NAE, NUtE, and HI values.

Fertilization Treatments	Row Spacing	NUE	NAE	NUtE	HI
2021					
C	D1	-	-	1.821 ± 0.036 a	99.705 ± 0.006 c
	D2	-	-	1.954 ± 0.025 A	99.681 ± 0.004 C
U	D1	0.073 ± 0.007 a	0.004 ± 0.0001 a	2.104 ± 0.037 b	99.654 ± 0.007 b
	D2	0.078 ± 0.006 A	0.005 ± 0.0003 A	2.273 ± 0.045 B	99.623 ± 0.008 B
I	D1	0.224 ± 0.005 b	0.010 ± 0.0004 b	2.377 ± 0.045 c	99.591 ± 0.008 a
	D2	0.260 ± 0.013 B	0.011 ± 0.0003 B	2.462 ± 0.018 C	99.575 ± 0.003 A
2022					
C	D1	-	-	2.202 ± 0.017 b	99.633 ± 0.002 b
	D2	-	-	2.236 ± 0.052 B	99.624 ± 0.009 B
U	D1	0.255 ± 0.010 a	0.005 ± 0.0006 a	2.158 ± 0.031 b	99.634 ± 0.005 b
	D2	0.247 ± 0.017 A	0.004 ± 0.0006 A	2.116 ± 0.014 A	99.638 ± 0.003 C
I	D1	0.455 ± 0.011 b	0.009 ± 0.0002 b	2.129 ± 0.009 a	99.631 ± 0.002 a
	D2	0.385 ± 0.015 B	0.008 ± 0.0008 B	2.219 ± 0.045 B	99.614 ± 0.008 A
2023					
C	D1	-	-	1.177 ± 0.008 a	99.794 ± 0.002 c
	D2	-	-	1.292 ± 0.031 A	99.772 ± 0.006 C
U	D1	0.129 ± 0.011 a	0.009 ± 0.0001 a	1.548 ± 0.012 b	99.727 ± 0.002 b
	D2	0.105 ± 0.009 A	0.008 ± 0.0008 A	1.610 ± 0.039 B	99.714 ± 0.007 B
I	D1	0.228 ± 0.011 b	0.015 ± 0.0007 b	1.798 ± 0.041 c	99.678 ± 0.008 a
	D2	0.232 ± 0.020 B	0.016 ± 0.0004 B	1.915 ± 0.013 C	99.652 ± 0.001 A
FYEAR (A)		353.99 ***	0.75 ns	4163.19 ***	3741.87 ***
FFERTILIZATION (B)		321.13 ***	74.44 ***	401.90 ***	479.14 ***
FROW SPACING (C)		23.49 ***	3.45 ns	31.40 ***	36.50 ***
FA × B		10.99 ***	9.80 **	167.86 ***	186.97 ***
FB × C		1.06 ns	0.06 ns	30.78 ***	28.65 ***
FA × C		27.81 ***	6.17 **	146.45 ***	144.84 ***
FA × B × C		8.77 **	42.44 ***	126.35 ***	118.73 ***

Means within a column followed by the different letters are significantly different at *p* = 0.05. Lower case letters a, b, and c correspond to statistically significant differences among fertilization treatments in D1, and uppercase letters A, B, and C correspond to statistically significant differences among fertilization treatments in D2. F-test ratios are from ANOVA, whereas ns, *, **, and *** correspond to no statistical significance, statistically significant at a level of *p* < 0.05, statistically significant at a level of *p* < 0.01, and statistically significant at a level of *p* < 0.001 respectively.

**Table 3:** Correlation matrix among agronomic traits, seed yield, N yield, N indicators, and NDVI and SPAD values.

	Year	Fertilization	Row Spacing	Plant Height	Fresh Weight	Dry Weight	Seed Yield	N Yield	NUE	NUtE	NAE	HI	SPAD 60 DAE	NDVI 60 DAE
Year	1.000	0.000 ns	0.000 ns	0.932 ***	0.936 ***	0.946 ***	0.258 ns	0.974 ***	0.282 *	−0.865 ***	0.019 ns	0.847 ***	−0.004 ns	0.043 ns
Fertilization		1.000	0.000 ns	0.328 **	0.324 **	0.278 **	0.593 ***	0.933 ***	0.787 ***	0.271 *	0.810 ***	−0.305 **	0.971 ***	0.307 **
Row spacing			1.000	0.097 ns	0.094 ns	0.086 ns	−0.036 ns	0.010 ns	−0.084 ns	0.054 ns	0.074 ns	−0.060 ns	0.170 ns	0.095 ns
Plant height				1.000	0.981 ***	0.987 ***	0.459 ***	0.942 ***	0.519 ***	−0.709 ***	0.296 *	0.678 ***	0.332 **	0.157 ns
Fresh weight					1.000	0.981 ***	0.411 **	0.964 ***	0.498 ***	−0.719 ***	0.279 *	0.691 ***	0.327 **	0.164 ns
Dry weight						1.000	0.434 **	0.959 ***	0.528 ***	−0.745 ***	0.266 ns	0.717 ***	0.281 *	0.151 ns
Seed yield							1.000	0.279 *	0.618 ***	0.086 ns	0.779 ***	−0.120 ns	0.572 ***	0.150 ns
N yield								1.000	0.390 *	−0.825 ***	0.142 ns	0.804 ***	0.228 *	0.296 *
NUE									1.000	−0.172 ns	0.702 ***	0.144 ns	0.743 ***	0.289 *
NUtE										1.000	0.309 *	−0.999 ***	0.288 *	−0.017 ns
NAE											1.000	−0.342 **	0.810 ***	0.204 ns
HI												1.000	−0.323 **	0.016 ns
SPAD 60 DAE													1.000	0.191 ns
NDVI 60 DAE														1.000

ns, *, **, and *** correspond to no statistical significance, statistically significant at a level of *p* < 0.05, statistically significant at a level of *p* < 0.01, and statistically significant at a level of *p* < 0.001 respectively.

**Table 4:** Soil properties of the studied experimental site.

Trait	Value
Soil Type	Clay Loam
Clay (%)	29.6
Silt (%)	34.6
Sand (%)	35.8
pH (1:1 H_2_O)	7.39
Organic Matter (%)	1.698
CaCO_3_ (%)	14.36
Total Nitrogen (%)	0.119
Phosphorus-Olsen P (mg kg^−1^ soil)	12.8
Potassium (mg kg^−1^ soil)	209

## Data Availability

The data presented in this study are available on request from the corresponding author.

## Abbreviations

The following abbreviations are used in this manuscript:

N	Nitrogen
C	Control
U	Urea
I	Urea with urease inhibitor
D1	Row spacing 30 cm × 20 cm
D2	Row spacing 50 cm × 20 cm
NDVI	Normalized Difference Vegetation Index
SPAD	Soil Plant Analysis Development
NUE	Nitrogen Use Efficiency
NUtE	Nitrogen Utilization Efficiency
NAE	Nitrogen Agronomic Efficiency
HI	Harvest Index
